# Characterization of a Stress-Enhanced Promoter from the Grass Halophyte, *Spartina alterniflora* L.

**DOI:** 10.3390/biology11121828

**Published:** 2022-12-15

**Authors:** Sonali Sengupta, Necla Pehlivan, Venkata Mangu, Kanniah Rajasekaran, Niranjan Baisakh

**Affiliations:** 1 School of Plant, Environmental and Soil Sciences, Louisiana State University Agricultural Center, Baton Rouge, LA 70803, USA; 2 Sanford Research, Sioux Falls, SD 57104, USA; 3 Department of Biological Sciences, Recep Tayyip Erdogan University, 53100 Rize, Turkey; 4 Plastomics Inc., Saint Louis, MO 63132, USA; 5 Southern Regional Research Center, New Orleans, LA 77054, USA

**Keywords:** abiotic stress, genetic engineering, halophyte, promoter, stress-inducible

## Abstract

**Simple Summary:**

Promoter is an important component to drive the expression of desirable genes in the genetic modification of plants. Constitutive promoters that direct the expression of genes at all times in all tissues may affect the growth and development of plants. Therefore, there is a need to increase the availability of promoters that are induced by different environmental stresses and/or developmental stages. Extremophiles, such as halophytes, which thrive under harsh environmental stresses, are considered important sources of such stress-inducible (or stress-enhanced) promoters. To this end, we isolated the promoter region of a stress-responsive gene *SaAsr1* from a grass halophyte smooth cordgrass. Characterization of the promoter and its deletion derivatives identified several regulatory elements that contribute to the expression of a gene at varying degrees under salt and drought stress. A known stress-responsive gene expressed under the control of the stress-enhanced promoter reported here produced plants that were normal and healthy in comparison with constitutive promoters that produced plants with compromised growth. Our results further validated the reports that gene expression under stress-inducible/enhanced promoter is a better strategy for genetic engineering to develop stress-resilient crop plants.

**Abstract:**

Stress-inducible promoters are vital for the desirable expression of genes, especially transcription factors, which could otherwise compromise growth and development when constitutively overexpressed in plants. Here, we report on the characterization of the promoter region of a stress-responsive gene *SaAsr1* from monocot halophyte cordgrass (*Spartina alterniflora*). Several *cis*-acting elements, such as ABRE (ABA-responsive element), DRE-CRT (dehydration responsive-element/C-Repeat), LTRE (low temperature-responsive element), ERE (ethylene-responsive element), LRE (light-responsive element), etc. contributed at varying degrees to salt-, drought- and ABA-enhanced expression of *gusA* reporter gene in *Arabidopsis thaliana* under the full-length promoter, pAsr1_1875_ and its deletion derivatives with an assortment of *cis*-regulatory motifs. The smallest promoter, pAsr1_491_, with three *cis*-acting elements (a CCAAT box-heat responsive, an LRE, and a copper responsive element) conferred drought-enhanced expression of *gusA*; pAsr1_755_ (with an ABRE and a DRE) presented the highest expression in ABA and drought; and pAsr1_994_ with seven ABREs and two DREs conferred optimal induction of *gusA*, especially under drought and ABA. *Arabidopsis* transgenics expressing a known abiotic stress-responsive gene, *SaADF2* (actin depolymerization factor 2), under both pAsr1_1875_ and p35S promoters outperformed the wild type (WT) with enhanced drought and salt tolerance contributed by higher relative water content and membrane stability with no significant difference between pAsr1_1875_:*SaADF2* or p35S:*SaADF2* lines. However, pAsr1_1875_:*SaADF2* lines produced healthy plants with robust shoot systems under salt stress and control compared to slightly stunted growth of the p35S:*SaADF2* plants. This reestablished the evidence that transgene expression under a stress-inducible promoter is a better strategy for the genetic manipulation of crops.

## 1. Introduction

Recombinant DNA technology (or genetic engineering), which involves (over)expression of a native or foreign gene (transgene), has allowed significant progress in the development of improved crops, including crops with enhanced resistance against various (a)biotic stresses. For functional expression of the transgene in plants, it must be under the control of a promoter, which contains binding sites for recognition by RNA polymerase to initiate and regulate transcription of the gene, the first and foremost step of gene expression. Promoter deployment strategy plays an important role in designing an optimal cassette for controlled expression of the gene(s) of interest in heterologous organisms.

Over the years, different types of promoters, such as constitutive, tissue-specific, inducible, viral and synthetic, have been isolated from a wide variety of organisms and used to develop genetically engineered plant systems [1,2]. A constitutive promoter drives gene expression in all cell types of a plant at all developmental stages. Such promoters are more useful when global expression of the gene(s) of interest is needed. The most commonly used and preferred constitutive promoter, CaMV35S, is derived from the cauliflower mosaic virus [3]. Due to the limited availability of promoters driving desired gene expression, the CaMV35S promoter is also most frequently used to drive the expression of selectable marker genes in addition to target genes in a single vector, which leads to a potential increase in homologous-dependent gene silencing [4] as well as public perception concern due to their viral origin [5].

The sessile lifestyle of plants necessitates the deployment of several strategies to adapt to various environmental stresses at the whole plant, tissue, organ and cell levels. The abiotic stress response is largely manifested by the coordinated expression of a cascade of genes involved in various stress-responsive networks. Because of the multigenic nature of abiotic stress tolerance traits, conventional breeding of stress-resistant crops has been slow. Genetic engineering, in addition to efficiently and precisely introducing stress tolerance genes into target plants, can help in understanding the regulation of genes to unravel the fine controls of stress tolerance traits [6]. Most stress-resistance genes have been expressed in plants under the control of the CaMV35S promoter, its derivatives and other constitutive promoters [7]. The use of constitutive plant promoters without substantial or little response to environmental conditions and/or developmental stages often fail to provide bioengineered plants their full yield potential due to complex interaction pattern between gene expression and environment/phenology. Moreover, expressing stress resistance genes under strong constitutive promoters may cost the fitness of transgenics due to the metabolic burden as demonstrated by rice lines overexpressing *DREB1a*, saltol zinc finger protein (STZ/ZAT10) and wax production 1 [8]. Rice transgenic lines overexpressing *OsLEA3-1* [9] or *OsSAP8* [10] display significantly higher yield or better physiological performance under stress than wild type (WT) but with compromised yield (up to ~50% yield reduction) under unstressed control conditions. To circumvent these problems, constitutive promoters of plant origin derived from highly active housekeeping genes (*ubiquitin, actin, tubulin and elf*—eukaryotic initiation factor) have been used in transgenic plant development. However, their application is limited due to their typical long size and low strength in directing gene expression.

Successful application of transgenic technology for stress resistance can be improved by the availability of efficacious broad-spectrum promoters guiding spatio-temporal expression patterns of transgenes [2]. Inducible promoters offer precise regulation of gene expression where desirable expression of the transgene can be achieved by endogenous or exogenous stimuli such as hormones, biotic and abiotic stresses and steroids [11]. Various promoters responsive to biotic/abiotic stresses or guiding tissue-specific expression patterns can be very useful for the generation of stress-resistant plants. The type, length and *cis*- and *trans*-acting factors of the promoters associated with the control of RNA polymerase expression play an important role in the regulation of plant gene expression, which also depends on other factors such as cell growth and developmental stages. Intensive efforts have been made to characterize the molecular structures of promoters and their effect on transcriptional regulation. The *cis*-regulatory elements in the promoter regions contain binding sites for transcription factors (TF). Characterization of the stress-specific *cis*-regulatory elements such as *DRE* (dehydration-responsive element; [12], cold-responsive *CBF* (C-repeat binding factor; [13], *ABRE* (ABA-responsive element, [14] in promoters has facilitated in designing stress-inducible promoters with superior efficacy.

Several stress-inducible promoters have been isolated from various non(crop) plants and used in transgenic plant research. Several studies showed that some superior and/or novel stress-tolerance mechanisms are unique to extremophiles such as halophytes [15], which suggests that the identification of stress-inducible promoters must be explored in extremophiles for successful exploitation of genes for stress tolerance [16]. Stress-responsive promoter regions of genes of extremophiles such as *Aeluropus littoralis*, *Ammopiptanthus mongolicust*, *Atriplex centralasiatica*, *Avicennia marina*, *Dunaliella salina*, *Mesembryanthemum crystallinum*, *Salicornia brachiata*, *Suaeda liaotungensis*, *Thellungiella halophila*, *Xerophyta viscosa* etc. have been isolated, characterized and demonstrated to exhibit strong transgene expression under stress with only a very few used for agricultural applications [15,17]. Therefore, it is important to isolate and exploit promoters from extremophiles that can drive manyfold higher expression of the genes under salt and/or drought stress without metabolic taxation and maintenance of basal expression under control conditions.

In this study, we describe the isolation and characterization of a promoter driving the expression of a stress-inducible gene, abscisic stress ripening (*SaAsr1*), from a monocot halophyte smooth cordgrass (*Spartina alterniflora*). We show that the promoter is enriched in several abiotic stress-related *cis*-acting elements. Through deletion series experiments using the *gusA* gene, we demonstrate that an assortment of promoter motifs leads to variation in gene expression under stress. Further, transgenic *Arabidopsis* plants expressing *SaADF2,* a stress-inducible actin depolymerizing factor from *S. alterniflora* [18,19] under the control of pAsr1_1875_ produced healthy plants with robust shoot systems under salt stress and control compared to p35S:*SaADF2*.

## 2. Materials and Methods

### 2.1. Isolation of the Promoter Region and in Silico Analysis of pASr1_1875_

An 1875-bp fragment including the 5′ UTR upstream of the abscisic stress-ripening 1 (*Asr1*) gene of *Spartina alterniflora* (NCBI Acc. # EH276806.1; [20] was isolated from the genomic DNA by inverse PCR-based genome walking as described earlier [21].

The full-length putative promoter pAsr1_1875_ was scanned for the presence of cis-regulatory motifs using PLACE [22] and PlantCARE [23]. Transcription factor binding sites were identified using a matinspector and further categorized into different classes based on their sequence similarity.

### 2.2. Generation of the Promoter: gusA Construct for Transient GUS Expression Assay

For transient glucuronidase (GUS) assay, the putative promoter pAsr1_1875_ was subcloned at *Hind*III-*Nco*I site of pCAMBIA0305.2 replacing 35S promoter upstream of the *GusPlus^TM^* gene following the standard cloning procedure as described earlier [21]. Callus was generated from mature seeds of rice cv “Cocodrie,” and 2-week-old calli were co-cultivated with *Agrobacterium tumefaciens* strain LBA4404 harboring the recombinant plasmid as described earlier [21]. After 3d of cocultivation, transient GUS expression was verified by incubating the calli in an X-gluc staining solution (50 mM Na_2_HPO_4_ buffer, pH 7.0, 0.1% Triton-X-100 and 0.5 mg/mL 5-bromo-4-chloro-3-indolyl b-D-glucuronide cyclohexylammonium salt) at 37 °C overnight.

### 2.3. Generation of the Full-Length and Deletion Series Promoter: gusA Constructs and Binary Vector with SaADF2 under the Control of pAsr1_1875_

**Figure 1:**
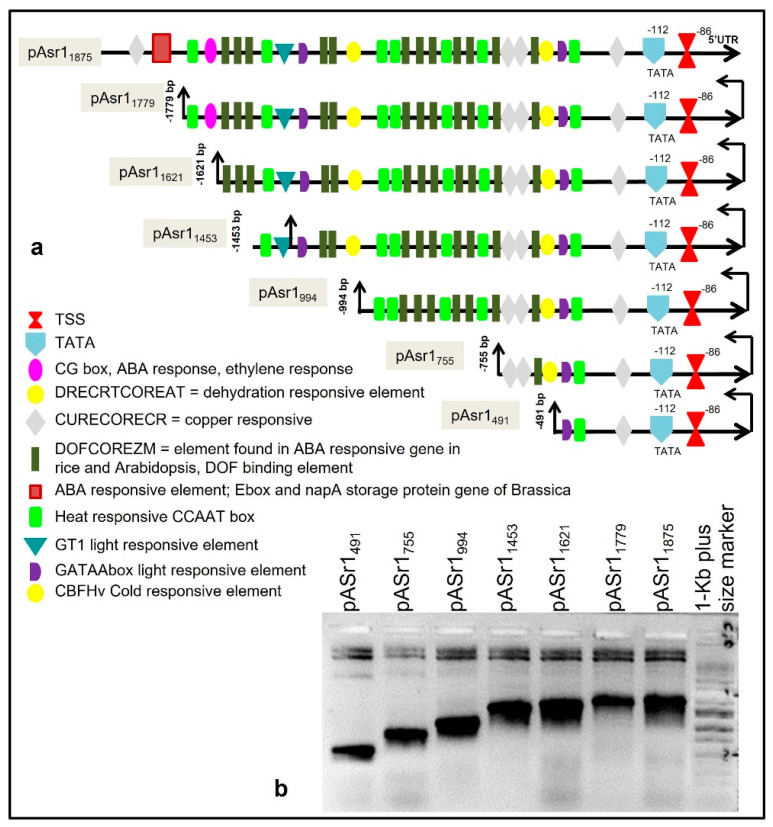
The arrangement of cis-acting motifs on pAsr11875 promoter and their functions (**a**). Several ABA-responsive and dehydration-responsive elements are overrepresented in the promoter. The positions of deletion mutants are highlighted (−491 bp–−1779 bp). (**b**) Gel image showing amplified bands corresponding to promoter deletion fragments that were used to construct gusA fusion vectors.

The pAsr1_1875_ was subcloned upstream of the *gusA* gene, replacing the CaMV35S promoter at the *Hind*III-*Nco*I site in pCAMBIA1305.1. Six stepwise 5´ deletions were designed based on the positional abundance of *cis*-regulatory elements in pAsr1_1875_ at positions −1779, −1621, −1453, −994, −755 and −491 nt that are henceforth referred to as pAsr1_1779_, pAsr1_1621_, pAsr1_1453_, pAsr1_994_, pAsr1_755_ and pAsr1_491_, respectively (Figure 1). Primer sequences used for the generation of the constructs are listed in Supplementary Table S1.

An abiotic stress resistance gene *SaADF2* from *S. alterniflora* was subcloned in pCAMBIA2301 downstream of the pAsr1_1875_ to compare the performance of the pAsr_1875_ promoter with 35S promoter driving *SaADF2* in pCAMBIA1301 [19].

### 2.4. Arabidopsis Thaliana Transformation

The plant expression constructs pMDC111NB [24] with *gusA* under the control of maize ubiquitin promoter [25] was mobilized into *Agrobacterium tumefaciens* strain LBA4404 using a freeze-thaw method and used for floral dip-mediated transformation of *Arabidopsis thaliana* ecotype Columbia (Col-0) as described earlier [26]. Positive transformants (T_0_), selected with germination of T_0_ seeds under hygromycin selection, were advanced to T_2_ generation under 25 °C and 16 h/8 h light/dark. Seeds from independent T_2_ homozygous lines were randomly selected for further analysis.

### 2.5. Stress Treatment

At least 20–30 seed-derived homozygous plants from each independent T_2_ line expressing *gusA* under pAsr1_1875_ were grown on ½ MS solid medium. Two-week-old plants were transferred to ½ MS solid medium supplemented with either mannitol (equivalent to drought at −0.3 MPa), 10 µM ABA and 150 mM NaCl for 5 days for imposing desiccation and salt stress, respectively.

For salt and drought stress with the promoter comparison study, 20 homozygous plants containing pAsr1_1875_:*SaADF2*, p35S:*SaADF2* and WT plants were transferred to soil and irrigated with 1/10th strength Hoagland’s medium. Drought stress was imposed on one-month-old plants by withdrawing irrigation for a week, and salt stress was enforced by watering with Hoagland’s solution plus 150 mM NaCl at 2-day intervals for a week. Six plants of pAsr1_1875_:*SaADF2* and p35S:*SaADF2* and seven WT plants were used each for drought and salt stress treatments, whereas 6 plants from each of the 3 genotypes were used as control. Root and shoot growth after 1 week of stress were recorded. Fully expanded leaves from rosettes on 6 lines (three independent lines each for both promoters) and WT were sampled for determination of relative water content (RWC) and membrane stability index (MSI) as described earlier [26]. Briefly, fully expanded leaves were collected and weighed immediately [fresh weight (FW)]. For RWC, the leaves were immersed in double-distilled water (ddH_2_O), placed in the dark at 4 °C overnight and weighed after brief blot-drying [turgid weight (TW)]. Then, the leaves were dried at 60 °C for 24 h and weighed [dry weight (DW)]. RWC was estimated using the formula: RWC (%) = [(FW–DW)/(TW–DW)] × 100. For MSI, 100 mg of leaf samples were heated at 40 °C for 30 min in 10 mL of double-distilled water, and the electrical conductivity of the solution (C1) was recorded. The leaves were then boiled at 100 °C for 10 min, and conductivity (C2) was measured. MSI was calculated as: MSI = [1 − (C1/C2)] × 100. Results of root length, RWC and MSI were presented as a % increase or decrease in pAsr1_1875_:*SaADF2* or p35S:*SaADF2* relative to the WT.

### 2.6. Histochemical GUS Staining

Expression of the *gusA* gene in plant tissues was observed via the histochemical assay using a method modified after [27]. Briefly, three control or stressed plants from each transgenic line and WT were incubated in an X-gluc staining solution for 16 h at 37 °C. Chlorophyll and other pigments were removed by washing the plants in 70% ethanol, and plants were observed for blue color development. pUbi:*gusA* and WT Arabidopsis were used for comparison as the positive and negative controls, respectively.

### 2.7. MUG Fluorescence Assay for Quantitative Measurement of gusA Activity

MUG (4-Methylumbelliferyl-ß-d-glucuronide hydrate) fluorometric assay was performed to quantitatively measure the GUS protein expression following the method described by Gallagher [27]. Briefly, 100 mg tissues collected from the treated and control plants were homogenized in 100 µL of extraction buffer (50 mM Phosphate Buffer, pH 7.0, 10 mM DTT, 1 mM Na_2_EDTA, 0.1% SDS, 0.1% Triton X-100) followed by centrifugation for 15 min at 4 °C. A 0.5 mL aliquot of MUG assay buffer (1 mM MUG in extraction buffer) pre-warmed at 37 °C was added to 50 µL crude protein extract and incubated overnight at 37 °C. The reaction was stopped by adding 0.2 M Na_2_CO_3_ mix, and 100 µL of the reaction mix was transferred to a 96-well plate for fluorescence measurement in a microplate reader (Biotek Synergy, Santa Clara, CA, USA; Excitation 365 nM, Emission 460 nM). 4-MU was used as the standard for quantification.

### 2.8. Semi-Quantitative Reverse Transcription-Polymerase Chain Reaction (RT-PCR)

Transcription of the *gusA* gene under the control of the promoter(s) of interest was assessed with RT-PCR using the RNA isolated from the T_2_ homozygous plants treated with NaCl, ABA and/or mannitol as described under stress treatment. *Spartina alterniflora* plants were subjected to salt and drought stress, as described earlier [20]. Total RNA was isolated with Trizol (Invitrogen, Carlsbad, CA, USA), and first-strand cDNA was synthesized from 500 ng of total RNA using iScript cDNA synthesis kit (Biorad, Hercules, CA, USA) as described earlier (Joshi et al., 2013). (Semi)quantitative RT-PCR was performed to monitor the expression of *gusA, SaADF2*, and *SaAsr1* as described earlier [20]. *Arabidopsis* elongation factor gene (*AtElf1a*) was used for the normalization of gene expression, and relative expression was calculated using the 2^−ΔΔCt^ method.

## 3. Results

### 3.1. Isolation and In-Silico Analysis of the pAsr1_1875_ Promoter

The abscisic stress-ripening 1 gene (*SaAsr1*) was identified as one of the many genes upregulated under salt stress in *Spartina alterniflora* [20]. Both *SaAsr1a* and *SaAsr1b* showed upregulation under drought stress in the leaf but not in root tissue (Supplementary Figure S1), and an 1875 bp upstream fragment of *SaAsr1a* was isolated through primer walking. Transient expression of the *gus* gene in *Agrobacterium tumefaciens*-infiltrated rice calli (Supplementary Figure S2) confirmed the promoter property of the promoter pAsr1_1875_ driving expression of a transgene.

The transcription start site (TSS = +1) on the promoter was mapped to an adenine (A) residue 86 bp upstream of the start codon (ATG; Figure 1), and the nearest predicted TATA box was located 25 bp upstream of the TSS. In silico analysis identified several *cis*-regulatory motifs, such as DRE-CRT (Dehydration Response Element/C-Repeat), ABRE (ABA-responsive element), CBF (C-repeat binding factors), LTRE (low temperature-responsive element), ERE (ethylene-responsive element), LRE (light-responsive element) etc., were identified in the promoter (Figure 1, Table 1). Ninety different classes of *cis*-regulatory elements with different functions, conserved motifs such as TATA box and CAAT box, and general and essential enhancers of transcription were present in the promoter sequence. An abundance (11) of the DNA binding with one finger (DOF)COREZM *cis*-regulatory element, initially reported to occur in the core site required for binding of plant-specific DOF proteins in maize [28] (Yanagisawa and Schmidt,1999), was observed in the promoter. Fifteen E-box/NAPA elements, initially identified in *Brassica napa* and known to be involved in both ABA response and seed storage [29], were found on the sense strand. CG-Box motif, involved in both ABA and ethylene response, was also overrepresented in the promoter sequence. Three DRECRTCOREAT elements that are the core motif of DRE/CRT (dehydration-responsive element/C-repeat) element in Arabidopsis and in rice were present [30]. CCAAT boxes were also overrepresented, which are frequently found in the promoter of heat shock proteins and act cooperatively with HSE (heat shock elements) to increase the heat shock promoter activity [31,32,33]. Consensus GT-1 binding elements were present, which are frequently implicated in the expression of light-responsive genes [34]. In addition, the promoter sequence contained myb1 and myc1 binding sites, a GATA box and several other biotic stress-responsive motifs (Table 1). 

### 3.2. Stable Reporter Gene Expression in Arabidopsis Transgenic Lines

**Figure 2:**
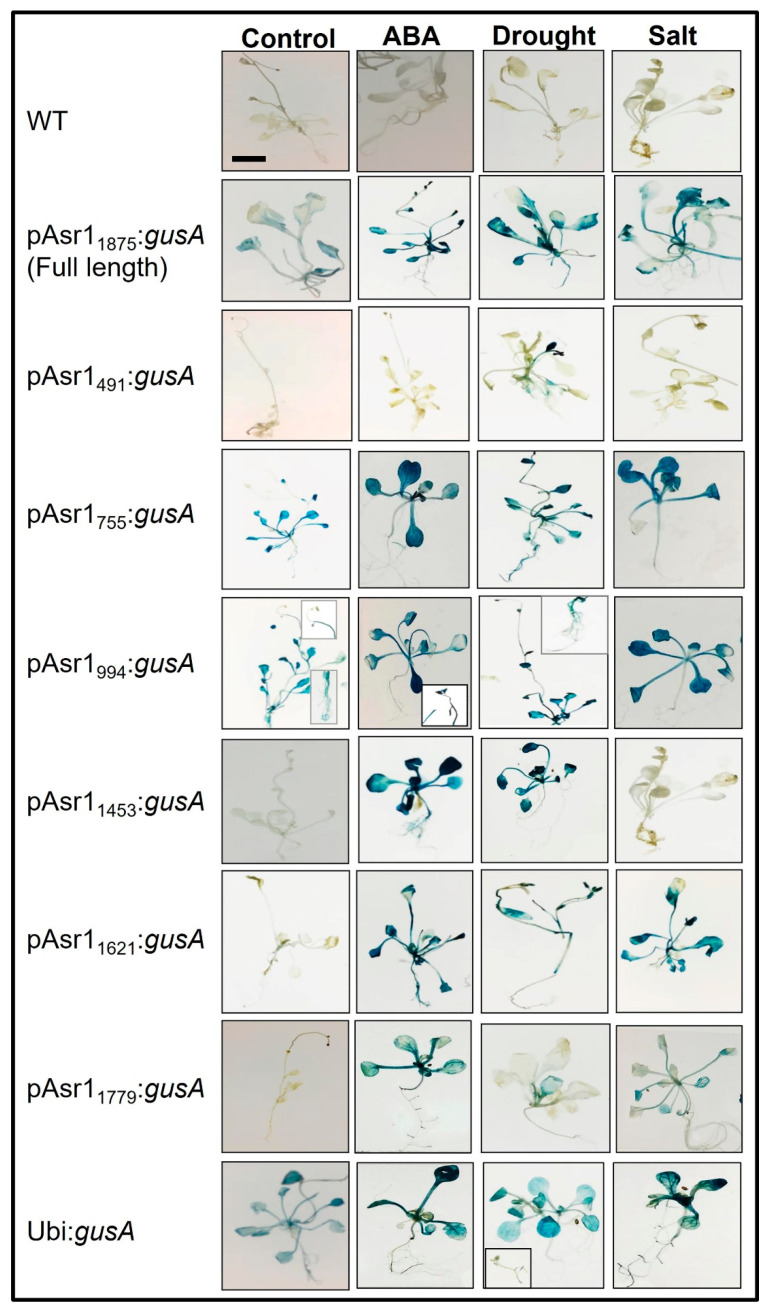
Histochemical GUS assay of Arabidopsis transgenics showing *gusA* gene expression under the control of pASR1 promoter constructs and constitutive maize ubiquitin promoter. Insets in plants with pAsr1_994_:*gusA* construct represent reproductive and root tissues at 10× magnification. Scale bar = 1.0 cm.

Differential expressions of the *gusA* gene were observed in the transgenic Arabidopsis plants carrying the promoter constructs, while no expression of the reporter gene was detected in the wild-type (WT, untransformed) plants. The expression level of *gusA* in the root, leaf and stem tissues varied with the combinatorial occurrence of *cis*-elements in the promoter, indicating that tissue-specific *cis*-elements are also present in the promoter (Figure 2). The smallest (491 nt) promoter of the series pAsr1_491_ containing three major *cis*-acting elements (a CCAAT box-heat responsive element, an LRE and a copper responsive element) showed low expression of *gusA* under unstressed control conditions but enhanced expression under stress with the highest induction in drought stress. The promoter pAsr1_755_ with one each of ABRE and DRE and pAsr1_994_ with seven ABREs and two DREs presented expression under control similar to the full-length promoter pAsr1_1875_, but much-enhanced induction in all stress conditions, especially ABA treatment and salt. pAsr1_1453_ exhibited minimal expression under control and salt but very high induction when treated with ABA and drought stress. Promoters pAsr1_1621_ and pAsr1_1779,_ each with 12 ABREs but five and six DREs, respectively, did not register *gusA* expression under control but showed induction under all stress conditions (Figure 2).

### 3.3. Quantitative MUG Assay and gusA Transcript Accumulation under Multiple Abiotic Stresses

**Figure 3:**
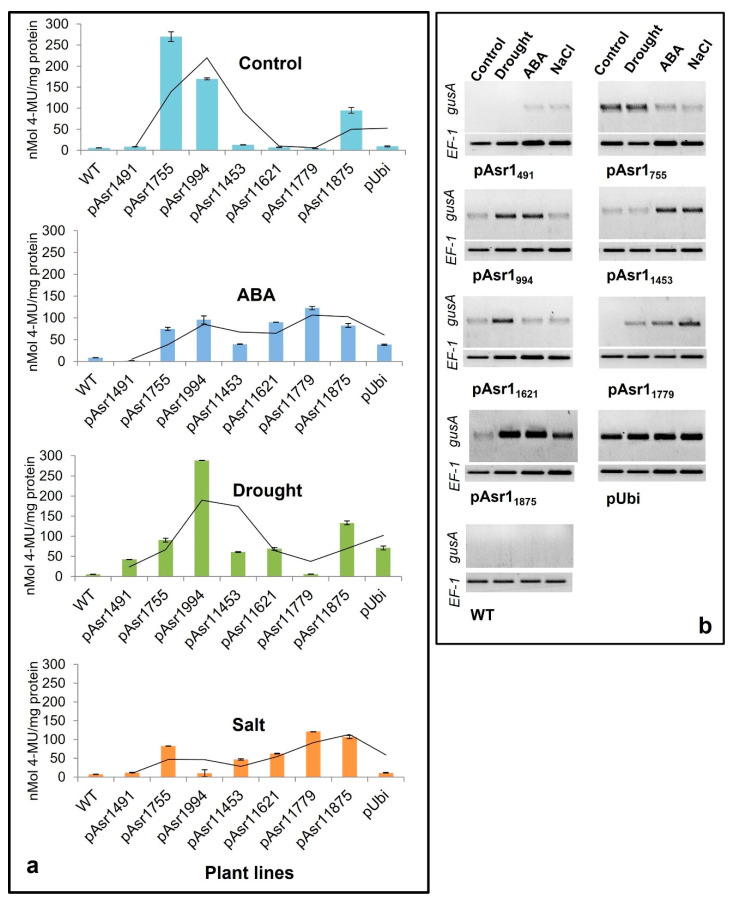
Quantitative MUG assay to measure GUS activity expressed as MU released/mg of total protein (**a**) and RT-PCR showing *gusA* transcripts (**b**) of Arabidopsis transgenics expressing the reporter gene driven by pASR1 promoter constructs and constitutive maize ubiquitin promoter under unstressed control and after a week of exposure to multiple abiotic stresses. The lines across data points in Figure 3a represent 2-period moving average trendlines.

Enzymatic MUG assay performed on Arabidopsis plants with the full-length and deletion promoter constructs showed significant changes in GUS protein depending on the presence/absence of *cis* elements in the promoter fragment (Figure 3a). Except for pAsr1_755_, pAsr1_994_ and full-length pAsr1_1875_, no other promoter deletions resulted in detectable GUS accumulation in the leaf under control conditions. On the other hand, all promoters except pAsr1_755_ showed higher GUS expression under all stresses. pAsr1_491_ responded slightly only to drought stress with enhanced GUS expression, whereas pAsr1_994_ had the highest expression under drought and higher than pAsr1_1875_ full-length promoter. pAsr1_1779_ resulted in higher GUS protein only when the plants were exposed to ABA and salt stress. The full-length promoter pAsr1_1875_ showed expression in unstressed condition albeit lower than both pAsr1_755_ and pAsr1_994_ (Figure 3a).

Semiquantitative RT-PCR results mostly corroborated the MUG and GUS histochemical assays. The *gusA* transcript was expressed in all conditions when expressed under the control of pAsr1_1875_, but its expression was significantly upregulated under drought, ABA and salt stress. The reporter gene driven by all the pAsr1_1875_ promoter derivatives except pAsr1_491_ under drought showed a higher mRNA accumulation than when it was driven by the constitutive promoter p35S (Figure 3b). In addition to pAsr1_1875_, pAsr1_755_, pAsr1_994,_ and pAsr1_1621_ had the maximum upregulation of *gusA* under drought stress. Under 10 µM ABA, all constructs except pAsr1_755_ and pAsr1_1621_ showed a higher *gusA* expression compared to unstressed control, which was ~1.5–2-fold over the p35S constitutive promoter. In salt stress, the full-length promoter pAsr1_1875_ showed the highest expression, followed by pAsr1_1453_ and pAsr1_1779_ (Figure 3b).

### 3.4. Physiological and Phenotypic Responses of SaADF2 Driven by pAsr1_1875_ and pCaMV35S under Salt and Drought Stress

**Figure 4:**
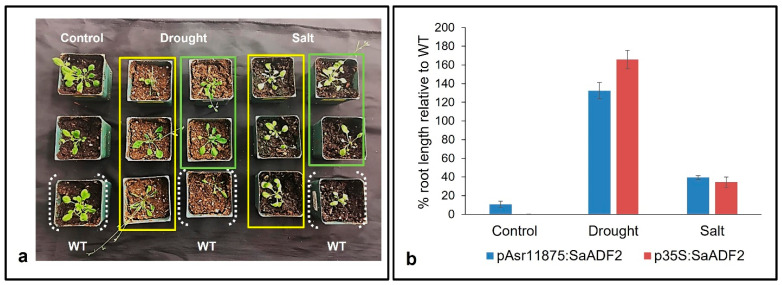
Phenotype (**a**) and percentage increase of root length relative to wild-type (WT) (**b**) of Arabidopsis mutants overexpressing SaADF2 gene under pAsr11875 (within the yellow rectangle) and constitutive p35S promoter (within the light green rectangle) after 1 week of exposure to drought stress and salt (150 mM NaCl) stress.

Soil stress experiments showed that stable T_2_ Arabidopsis transgenics expressing *SaADF2* under p35S as well as pAsr1_1875_ promoter showed better phenotypic response with improved growth under salt and drought stress-induced osmotic stress compared to WT Arabidopsis (Figure 4a). Plants from all lines could grow to some extent under both stresses, but the WT plants became bleached and rolled (leaf rolling) by the 7th day of salt and drought stress, respectively, while rosettes of plants with either pAsr1_1875_:*SaADF2* or p35S:*SaADF2* were less affected under stress with green and turgid leaves in comparison with WT (Figure 4a). Under non-stress control conditions, the growth of all plants was similar, although the transgenics with SaADF2 under p35S were a little stunted in growth. The average root length of transgenics with *SaADF2* under pAsr1_1875_ was slightly higher (10.8%; 1.72 cm) compared to 1.54 cm for the WT and plants expressing *SaADF2* under p35S. Under stress, on the other hand, pAsr1_1875_:*SaADF2* and p35S:*SaADF2* transgenics recorded 39% and 34% higher root length under salt and 132% and 165% higher root length under drought, respectively, than WT (Figure 4b).

**Figure 5:**
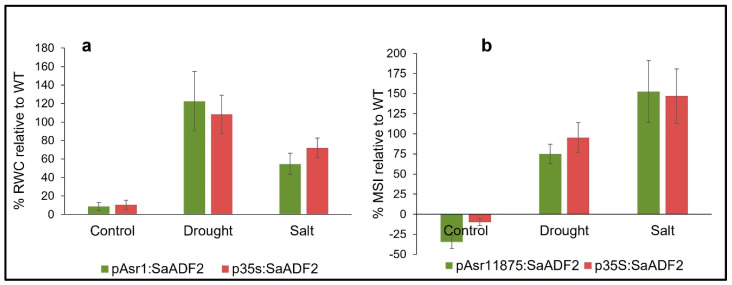
Physiological response expressed as percentage increase in relative water content (RWC; (**a**)) and membrane stability index (MSI; (**b**)) of Arabidopsis mutants overexpressing *SaADF2* gene under pAsr1_1875_ and constitutive p35S promoter relative to wild-type (WT) after 1 week of exposure to drought stress and salt (150 mM NaCl) stress.

Arabidopsis plants with *SaADF2* under both promoters showed higher relative water content (RWC) and membrane stability index (MSI) than the WT under salt and drought stress. Under the control condition, the transgenics had a slightly higher (8–10%) RWC relative to the WT (Figure 5a). On average, p35S:*SaADF2* plants performed better with 72% higher RWC than WT as compared to 55% for pAsr1_1875_:*SaADF2* plants under salt stress. Under drought stress, however, pAsr1_1875_:*SaADF2* plants had 122% higher RWC compared to 108% for p35S:*SaADF2* relative to WT. Interestingly, MSI was found to be slightly higher in WT (1.42) relative to plants with p35S:*SaADF2* (1.3; −10.0%) and pAsr1_1875_:*SaADF2* (0.94; −34.9%) under unstressed condition (Figure 5b). But transgenics expressing *SaADF2* driven by both pAsr1_1875_ and p35S outperformed with 75.0% and 95.3% higher, respectively, under salt stress and 152.4% and 147.0% higher, respectively, relative to WT under drought stress.

## 4. Discussion

A wider range of effective and efficient promoter elements would facilitate the introduction of multiple transgenes in plant cells minimizing the risk of homology-dependent gene silencing. Bioengineering of plants for improved stress tolerance requires stress-inducible/enhanced promoters for controlled, yet high-level expression of genes associated with stress resistance mechanisms, which will have wide spectrum applications from exploratory research to agricultural field practices. To this end, current advancement in genomics has enabled the identification of new plant promoters for targeted expression of genes in a spatio-temporal and environment-dependent manner.

Under abiotic stress, gene expression is mostly regulated through ABA-dependent and/or ABA-independent pathways, with significant cross-talk between the two transcriptional control pathways [35]. Among various transcriptional regulators, the key *cis*-and *trans*-elements recognized in these two important pathways are (a) ABA-responsive element (ABRE) functioning together with ABRE binding transcription factors (ABFs) and (b) dehydration-responsive element/C-repeat (DRE/CRT) along with dehydration-responsive element binding transcription factors (DREBs). In addition to ABRE, the ABA-dependent pathway includes MYB2/MYC2 binding to MYBRS/MYCRS and NAC TF binding to CNAC elements, whereas the ABA-independent transcriptional pathway also includes NAC and HD-ZIP TF involved in ERD1 (early response to dehydration 1) gene expression. Osmotic adjustment and ROS scavenging generally function through ABA-dependent pathways, whereas ABA-independent pathways regulate the protection of proteins and organellar membranes.

The present study focused on the characterization of a promoter sequence of abscisic stress ripening gene 1 (*SaAsr1*) from the salt marsh cordgrass *Spartina alterniflora*. Promoter scanning of the *SaAsr1* gene showed an overrepresentation of several abiotic stress-related motifs, including motifs important for both ABA-dependent and ABA-independent pathways. The position and functions of the *cis*-regulatory elements are detailed in Table 1 and Supplementary Table S2. Of the total 90 TF binding domains present in the promoter, 39 for the Arabidopsis homeobox protein, the major TF-binding families related to osmotic stress, 21 for MYB TF, 14 for MYB-like proteins, 12 for NAC protein and 11 motifs each for DOF and GTBox proteins were overrepresented (Supplementary Table S2). The presence of seven DREB elements and one ABRE in the promoter indicated possible interactions between ABA-dependent and ABA-independent pathways and that the promoter could prove useful at both the early and late stages of dehydration [36].

Although homology search identified several *cis*-regulatory motifs in the full-length promoter pAsr1_1875_, the functionality of the motifs is dependent on several factors, including the presence of appropriate transcription factors and accessory proteins, tissue type, the presence of repressor elements and occasional regulators at the post-transcriptional level. The smallest promoter construct, pAsr1_491,_ contained two major groups of *cis*-regulatory elements: a heat-responsive CCAAT box and a light-responsive GATAA box element, and it showed only mild expression of the *gusA* reporter gene under drought. On the other hand, pAsr1_755_ with one DOFCOREZM (ABA-responsive) and one DRE element in excess showed GUS expression in unstressed as well as all stressed conditions, while pAsr1_994_ with four CCAAT boxes and six DOFCOREZM motifs drove a very high expression under drought and ABA, as well as in unstressed condition. There was a general decline in the activity of the promoter elements –994–1779 bp upstream under unstressed conditions. This could be due to the presence of three transcriptional repressors, BELLRINGER (–1203 bp), AS1/AS2 repressor complex binding motif II (–1103 bp), and KANNADI 4 (–1353 bp) in the –1099 bp–1359 bp region. However, the construct pAsr1_1621_ was highly upregulated under stress compared to the unstressed condition, probably because of the presence of five DOFCOREZM motifs in this region. pAsr1_1779_, the construct that harbors the CG box and is 106 bp shorter than the full-length promoter, showed a high expression following ABA treatment. This effect was further strengthened in the full-length promoter pAsr1_1875_, where the EboxNAPA element possibly was the major enhancer of its stress induction, despite the presence of a repressor SBEF at –1830 bp. Several previous studies have shown that the presence of these *cis*-regulatory elements is associated with the response to dehydration and/or abiotic stresses [36,37,38,39]. The other constructs did not actively express GUS under control conditions, indicating the presence of repressor elements in them. The results accord to the numerous reports that the frequency and spatial organization of the *cis-*regulating elements in the promoters potentially resulted in the differential activity of the promoter fragments in driving *gusA* expression in different tissues/stresses of transgenics. The inconsistent activity of the deletion promoters observed in the present study could be due to the heterologous Arabidopsis system used for reporter gene expression study instead of native *Spartina alterniflora*.

We have previously shown that *SaADF2* is a highly efficient actin depolymerizing factor that provides enhanced drought and salt tolerance in both rice and Arabidopsis [18,19]. Therefore, the results, expectedly, showed no significant difference in the efficiency between the two promoters, pAsr1_1875_ and p35S. Arabidopsis transgenics overexpressing *SaADF2* under both pAsr1_1875_ and p35S showed improved phenotypic and physiological response under salt and drought stress by maintaining higher root growth, relative water content and membrane stability index. However, the pAsr1_1875_ promoter had a comparative advantage over p35S for relative water content under drought stress, where the pAsr1:*SaADF2* transgenics maintained 122% more RWC relative to WT compared to 108% for p35S:*SaADF2*. Under salt stress, Arabidopsis lines expressing *SaADF2* under pAsr1_1875_ had a slightly higher membrane stability index than the p35S:*SaADF2* relative to WT. In general, early seedling vigor and aboveground growth of the transgenics expressing the gene under pAsr1_1875_ was better compared to p35S. The results suggested that the presence of the differential *cis*-regulating elements in pAsr1_1875_ contributed to an enhanced yet controlled (balanced) expression of *SaADF2* to confer the transgenics their superior performance, especially in drought stress.

## 5. Conclusions

The use of pAsr1_1875_ to drive the expression of a known stress-resistance-related gene *SaADF2* further strengthened the claim that the use of plant-origin stress-enhanced promoter provides better agronomic performance over constitutive promoters without compromising the stress tolerance functionality of the gene. These observations put forward pAsr1_1875_ and some of its derivatives as strong candidates among extremophilic plant-derived promoters to be used in abiotic stress bioengineering for expression of stress-responsive genes, especially the transcription factors, to offset the phenotypic penalty often associated with expressing the genes under constitutive promoters. The dissection of *cis*-regulatory elements via different combinations showed that it is possible to achieve a very high stress-induced expression of the gene of interest by using deletion constructs pAsr1_755_ and pAsr1_994_ if the low basal expression of the gene does not compromise the growth and development of plants_._ On the other hand, derivatives such as pAsr1_1453_, pAsr1_1621_ and pAsr1_1779_ could be used mostly as stress-inducible promoters depending on the stress the plant is exposed to. This opens the further possibility to design synthetic promoters by incorporating *cis*-regulatory elements (such as ABREs and DREs) to minimal promoter background to modulate its stress-specific regulation.

## Figures and Tables

**Table 1:** Stress-associated *cis*-element families present on the promoter sequence, their position and motif sequence.

Matrix Information	Anchor Position (+)	Sequence
R2R3-type myb-like transcription factor (I-type binding site)	93	gttcgtgcCAGTtgccaattt
Calmodulin-binding NAC protein	152	ctttGCTTtttctttgtgctt
Myb domain protein 99 (ATMYBCU15)	165	ttgtgcttgagcTAGGtgctc
Myb domain protein 46	169	gcttgagcTAGGtgctcccta
Homeobox protein 34	209	tatcttttTAATgaaatac
Homeodomain GLABROUS 1	209	atctttttAATGaaata
Calmodulin-binding transcription activator 1 (AtSR2)	223	ataCGCGctcatatgtg
MADS-box protein SQUAMOSA	243	gttcgctaaAAAAagtttttc
KH and zinc finger CCCH domain-containing protein	245	aaaaAAAGttt
Dof zinc finger protein DOF5.4 (OBF binding protein 4)	246	tcgctaaaaAAAGtttttcgggg
NAC WITH TRANSMEMBRANE MOTIF 1-LIKE 8	275	agttctcacaagtagaGGAAagg
DOF Affecting Germination 2	300	gagacaaatAAAGttggaacatt
Heat shock transcription factor C1	330	atctcgttttCAAGaagctatta
NAC domain-containing protein 92 (NAC6)	330	acatctcgttttcaaGAAGctattaag
AP2/ERF and B3 domain-containing transcription factor RAV1	380	atcAACAcagatt
GT1-Box binding factors with a trihelix DNA-binding domain	402	ttgtgtgtgGTTAcaatag
Myb domain protein 96 (MYBCOV1)	402	tttgtgtgtGGTTacaataga
Early Flowering MYB Protein (AT2G03500)	462	ttgaatATTCtaa
Heat shock transcription factor B2A	466	cttgaataTTCTaaattagcatc
I-Box in rbcS genes and other light-regulated genes	482	catcaTATAagaatatc
MYB protein from wheat	500	tgtcatATATtccttgtca
Auxin Response Element	508	cctTGTCacactt
Myb domain protein 33	539	gtgcgtttcaGTTTcaggagg
Homeobox-leucine zipper protein ATHB-24	555	gagggaaataATTAataaa
Homeobox protein 34	559	gaaataatTAATaaaaaat
Homeodomain GLABROUS 1	559	aaataattAATAaaaaa
Homeobox-leucine zipper protein ATHB-15 (INCURVATA 4)	579	acacataATGAtactcgag
Homeobox-leucine zipper protein ATHB-5	618	aaataGAATaattccacat
ABA response elements	625	taattccACATgtcagt
Myb domain protein r1 (ATMYB44)	632	ccacatgtcaGTTAtcctaat
MybSt1 (Myb Solanum tuberosum 1) with a single myb repeat	636	tgtcagttATCCtaataag
Homeobox 51, Late Meristem Identity 1	671	aataataaTAATgaattct
Arabidopsis thaliana ZF6 (cold-induced zinc finger protein 2)	699	gtaaCACTatc
Myb domain protein 107	740	gaatgaacagagTTGGttaag
MYB-responsive element, MYB46 and MYB83 binding sites	744	gaacagagTTGGttaagtgtc
Myb-like protein of Petunia hybrida	745	aacagagtTGGTtaagtgtct
Class I GATA factors	768	tcattGATAagacttaa
*Cis*-element in the GAPDH promoters conferring light inducibility	805	gcaaATAAagaggaa
Dof1/MNB1a—single zinc finger transcription factor	806	gatgcaaatAAAGaggaataaat
Myb family transcription factor REVEILLE 1	817	ggaataaaTATCttggt
Ethylene-responsive elements (ERE) and jasmonate- and elicitor-responsive elements (JERE)	833	gtttagagtCGCCgtatcg
Ethylene-responsive transcription factor RAP2-6 (secondary DNA binding preference)	865	ccaggcaGCCGacaccttc
DREB and EAR motif protein 4 (RAP2.10)	866	ccaggcagcCGACaccttctc
Auxin Response Element	889	gtgTGTCccaatt
Auxin Response Element	905	ccgTGTCaccatc
Myb family transcription factor At5g56840	920	ctttcctTTTCcaattgca
MybSt1 (Myb Solanum tuberosum 1) with a single myb repeat	952	ttacctttATCCaaagtta
Myb family transcription factor REVEILLE 1	963	aaagttaaTATCgatgg
NAC WITH TRANSMEMBRANE MOTIF 1-LIKE 8	968	agttaatatcgatggaGGAAaag
HD-ZIP class III protein ATHB9	979	ggaggaaAAGAttgcaaga
Calmodulin-binding NAC protein	999	ggttGCTTaagccacaaacaa
NAC domain-containing protein 87	1001	aggttgCTTAagccacaaacaacatca
Homeodomain protein WUSCHEL	1066	actgagATTAatatttctt
Oryza sativa CaM-binding transcription factor	1150	aatCGTGtactctaggc
Homeobox-leucine zipper protein ATHB-53	1221	ggataCAAAaatttagcac
Myb domain protein r1 (ATMYB44)	1271	gcttcatgcaGTTAtcttttt
Arabidopsis NAC domain-containing protein 19	1282	cagttatctttttttTACGgaacatgc
NACL-inducible gene 1	1286	tttTTACggaaca
NAC domain-containing protein 87	1313	atctttCTTTtttgacgcgaaagcagt
WRKY plant-specific zinc-finger-type factor; W box	1314	cttttTTGAcgcgaaag
Calmodulin-binding transcription activator 1 (AtSR2)	1320	tgaCGCGaaagcagtca
Arabidopsis thaliana ZF6 (cold-induced zinc finger protein 2)	1339	ctaaTACTaat
Myb family transcription factor At3g10113	1344	aatactAATAtccgaat
Myb family transcription factor At5g61620	1346	atactaaTATCcgaatatt
Myb family transcription factor (G2-like family)	1352	tatccgaatATTCcaag
MYB protein from wheat	1354	atccgaATATtccaaggat
Heat shock transcription factor B2A	1357	tccgaataTTCCaaggattatcc
Hordeum vulgare Myb-related CAB-promoter-binding protein 1	1366	caaggattATCCtctgccg
Ethylene-responsive transcription factor RAP2-6 (secondary DNA binding preference)	1374	atcctctGCCGacagttta
DREB and EAR motif protein 4 (RAP2.10)	1375	atcctctgcCGACagtttagc
NAC domain-containing protein 87	1415	ccatttCTTCgattagaggataaccaa
CCAAT-box in plant promoters	1427	aaCCAAtgg
Ethylene-responsive transcription factor RAP2-6 (secondary DNA binding preference)	1463	gggtcctGCCGtccaataa
Ethylene-responsive transcription factor ERF018	1464	gggtcctGCCGtccaataact
CCAAT-box in plant promoters	1468	gtCCAAtaa
Homeobox-leucine zipper protein ATHB-53	1471	ccgtcCAATaactgtccgg
Ethylene-responsive transcription factor RAP2-6 (secondary DNA binding preference)	1500	acttgatGCCGtcatgggc
Dehydration-responsive element-binding protein A-4	1501	acttgatgcCGTCatgggcag
NAC domain-containing protein 92 (NAC6)	1507	tgatgccgtcatgggCAGGcatccgtg
MybSt1 (myb Solanum tuberosum 1) with a single myb repeat	1576	gctccactATCCatcaatt
Homeobox-leucine zipper protein ATHB-23	1631	taatcgtttgATTAatctg
Homeodomain protein WUSCHEL	1635	cgtttgATTAatctgcaca
Homeobox protein 32	1665	caattttaTTATgtactgt
Hordeum vulgare Myb-related CAB-promoter-binding protein 1	1676	tgtactgtATCCtttgcta
Homeobox protein 33	1709	cagtgtgtTAATcacaatc
WUSCHEL-related homeobox 13	1716	tcaCAATcacc
Myb-domain transcription factor werewolf	1729	accctttgcaGTTTgcaccgt
Ethylene-responsive transcription factor ERF017 (AT1G19210)	1738	agtttgcACCGtccatcgatc
M-phase-specific activators (NtmybA1, NtmybA2, NtmybB)	1829	cgtccAACTgtcactgctgtc
Basic leucine-zipper 52	1841	gctgtCAGCtctcaa

## Data Availability

Data supporting the results are presented within the manuscript and as supplementary materials.

## Supplementary Materials

The following supporting information can be downloaded at: https://www.mdpi.com/article/10.3390/biology11121828/s1. Figure S1: RT-PCR showing expression of *SaAsr1* gene in leaf (L) and root (R) tissues Spartina alterniflora under control (c) and drought (d) and salt (s) stress. B, blank; M, 1-kb plus DNA size marker. Figure S2: Validation of promoter activity of pAsr1_1875_ fragment. Blue coloration indicates transient expression of the *gusA* reporter gene in rice callus co-cultivated with Agrobacterium tumefaciens carrying pAsr1_1875_:*gusA* construct (center and right panel) while the non-transformed WT (left panel) callus do not show any GUS activity. Insets, 10 × magnification. Table S1: Sequence of the primers used for generation of deletion series promoter constructs. Table S2: Overrepresentation of the transcription factor (TF)-family binding domains on pAsr1_1875_ promoter and their functional category.
