# Supplementary material for: Characterization of a Stress-Enhanced Promoter from the Grass Halophyte, Spartina alterniflora L
Source: Biology (Basel). 2022 Dec 15;11(12):1828. doi: 10.3390/biology11121828 (PMC9775435; doi:10.3390/biology11121828)
Supplement: Supplementary file 1 [file biology-11-01828-s001.zip › biology-2104897-supplementary.pdf]

Supplementary Table S1. Primers used for generation of deletion series promoter constructs

Primer ID	Seq with restriction sites (5´- 3´)
pAsr1$_{1875}$ R	CATGCCATGGGGCCGGCCGGGACCGATCGGTG
pAsr1$_{1875}$ F	CCCAAGCTTGGTGCGTGAAGATGGAGGTACCTTG
pAsr1$_{1779}$ F	CCCAAGCTTCCAATTTGTGGTCTCGTGTTTTCATGCTTTG
pAsr1$_{1621}$ F	CCCAAGCTTCATATGTGTGTTCGCTAAAAAAAGTTTTTCGG
pAsr1$_{1453}$ F	CCCAAGCTTGTTTGTGTGTGGTTACAATAGATGGATGTAGC
pAsr1$_{994}$F	CCCAAGCTTCGTGTGTCCCAATTGCACCGTGTCAC
pAsr1$_{755}$F	CCCAAGCTTTTGCAGACGCTGATAGGAAAAAATCG
pAsr1$_{491}$F	CCCAAGCTTCTCATCTGCTCATGCTTCCATTTCTTCG

Supplementary Table S2. Overrepresentation of TF-family binding domains and their functional category

TF Family	Match	Functional category
Arabidopsis homeobox protein	39	ABA responsive
MYB proteins with single DNA binding repeat	21	Water and salt stress
Circadian control factors	14	Auxin, salt stress
L1 box, motif for L1 layer-specific expression	14	Morphogenesis
MYB-like proteins	14	Water and salt stress
Plant specific NAC	12	Negative regulation of ABA response
DNA binding with one finger (DOF)	11	Response to water stimulus
GT-box elements	11	cellular response to water deprivation
Transcription repressor KANADI	11	Transcriptional repressor
Plant I-Box sites	8	Light response
MADS box proteins	8	Flowering ABA, Cold
NAC factors with transmembrane motif	8	Cold and salt stress
Vertebrate TATA binding protein factor	7	Transcriptional core element
Dehydration responsive element binding factors	7	Dehydration, cold, salt
Heat shock factors	7	Heat, light, hypoxia
Nodulin consensus sequence 1	7	Nodule induction
DNA-binding proteins with plant-specific TCP-domain	7	ABA and Auxin
Myc-like basic helix-loop-helix binding factors	6	Light response
NAC domain transcription factors	6	Drought recovery, reactive oxygen species
Storekeeper like transcriptional regulators	6	Negative regulation, sugar response
W Box family	6	ABA-activated signaling, salt stress, water deprivation

Auxin response element	5	Reproductive development
Calmodulin binding / CGCG box binding proteins	5	Cold response
GCC box family	5	salt, water stress
Golden2-like factors	5	phosphate starvation
LOB domain factors	5	Development
Legumin Box family	5	seed-specific
MYB IIG-type binding sites	5	Water deprivation, salt stress
Secondary wall NACs	5	Development
AT-hook containing transcription factors	4	Defense response
M-phase-specific activator elements	4	Negative regulation
Opaque-2 like transcriptional activators	4	cellular response to abscisic acid stimulus
Factors involved in programmed cell death response	4	Apoptosis
SBP-domain proteins	4	Anther-specific
SHI related sequence family members	4	Auxin homeostasis
CRC domain containing tesmin/TSO1-like CXC (TCX) factors	4	Ovule specific
Time-of-day-specific cis regulatory elements	4	Circadian rhythm
Core promoter motif ten elements	3	Core promoter element, TATA-independent
Yeast TATA binding protein factor	3	Transcriptional core element
AS1/AS2 repressor complex	3	Salt stress, defense, Repressor
Brassinosteroid (BR) response element	3	Brassinosteroid (BR) response
CCCH domain-containing factors	3	RNA processing
Calcium regulated NAC-factors	3	Negative regulation
GA- and ABA-responsive zinc finger like factors	3	ABA, GA
Jasmonate response element	3	JA, ABA, wounding, salt stress
LFY binding site	3	Flowering
Sucrose box	3	Sugar Response
Basic/leucine zipper-type transcription factors of the TGA-family - TGACG motif-binding factors	3	development
Zinc fingers of Arabidopsis thaliana	3	Phosphate ion homeostasis
Core promoter initiator elements	2	Transcription initiation
ABA response elements	2	abscisic acid-activated signaling pathway
Auxin Response Factor 3	2	Auxin metabolism, floral metabolism
CCAAT binding factors	2	ABA response, water stress
Ethylene respone element factors	2	morphogenesis
Flowering bHLH factors	2	Flowering, stomatal movement

GAP-Box (light response elements)	2	Light response
Plant G-box/C-box bZIP proteins	2	ABA, osmotic stress, salt stress, calcium
Growth regulating factor family	2	Leaf development
Light responsive element motif	2	Hypoxia
Conserved box A in PAL and 4CL gene promoters	2	Elicitor-responsive
Plant nitrate-responsive cis-elements	2	Nitrate assimilation, water deprivation
Pollen-specific regulatory elements	2	Pollen development
B3 domain-containing REM family	2	flower development
RWP-RK domain proteins	2	Nitrogen signaling
Root hair-specific cis-elements in angiosperms	2	Root-specific
Upstream sequence element of U-snRNA genes	2	RNA processing
WUS homeobox-containing protein family	2	Repressor in stem cell activator in floral patterning
General transcription factor IIIC, GTF3C	1	Core element
B-box type zinc finger-containing proteins	1	Core, ubiquitous
Dc3 promoter binding factors	1	Seed-specific, ABA responsive
E2F-homolog cell cycle regulators	1	DNA repair
Ethylen insensitive 3 like factors	1	Ethylene, Hypoxia, Sugar
EPF-type zinc finger factors	1	Defence
ER stress-response elements	1	ER stress
Floral homeotic protein APETALA 2	1	Reproductive
Myb-related DNA binding proteins (Golden2, ARR, Psr)	1	Floral development
High mobility group factors	1	Transcriptional enhancer
ID domain factors	1	Aux Biosynthesis
Mycorrhiza transcription factor	1	Elicitor
Nodulin consensus sequence 3	1	Nitrogen signaling
NACL-inducible genes	1	hyperosmotic salinity response
Paired amphipathic helix domain factors	1	Chromatin modulation
Protein secretory pathway element	1	Heat responsive
5'-part of bipartite RAV1 binding site	1	Not characterized
Soybean embryo factor 4	1	Seed specific
Transposase-derived transcription factors	1	Circadian Rhythm
TEF-box	1	Telomeric, activation
Telo box (plant interstitial telomere motifs)	1	response to salt stress, ethylene, SA
VIP1 responsive elements	1	Osmotic stress, salt stress, sulphate starvation
WT-Box	1	Circadian Rhythm

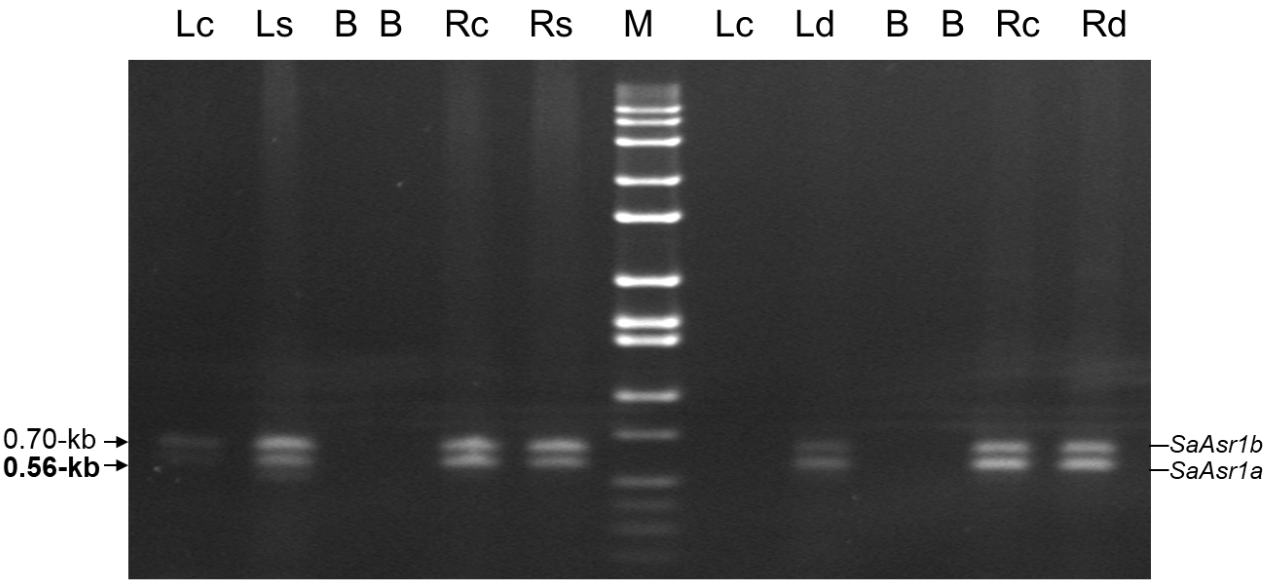

Supplementary Fig. S1

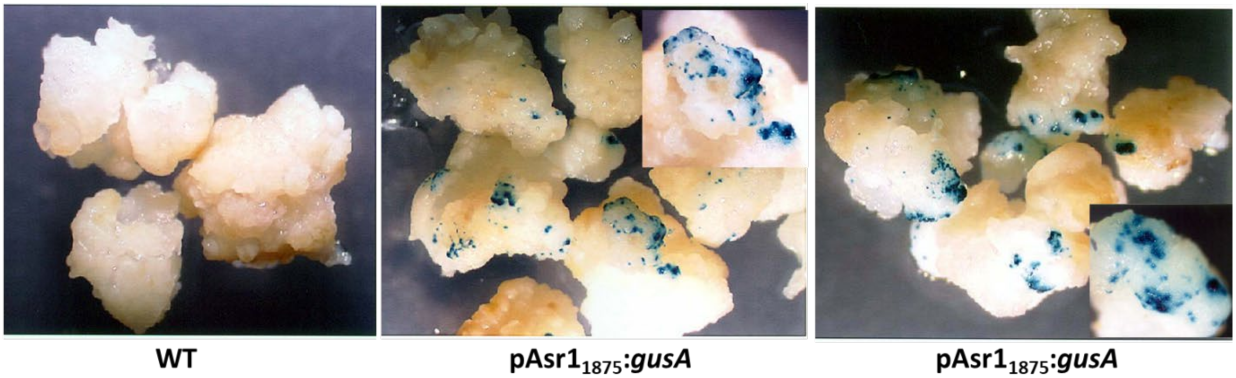

Supplementary Fig. S2
